# The effects of Autonomous Sensory Meridian Response (ASMR) videos versus walking tour videos on ASMR experience, positive affect and state relaxation

**DOI:** 10.1371/journal.pone.0277990

**Published:** 2023-01-04

**Authors:** Tobias Lohaus, Sara Yüksekdag, Silja Bellingrath, Patrizia Thoma

**Affiliations:** 1 Neuropsychological Therapy Centre (NTC), Faculty of Psychology, Ruhr University Bochum, Bochum, Germany; 2 Department of Cognitive Neuroscience, Faculty of Psychology and Neuroscience (FPN), Maastricht University, Maastricht, The Netherlands; 3 Institute of Psychology, Faculty of Educational Sciences, University Duisburg-Essen, Essen, Germany; Anglia Ruskin University, UNITED KINGDOM

## Abstract

**Objective:**

Autonomous Sensory Meridian Response (ASMR), the experience of a pleasant tingling on the neck and scalp, is known to be triggered by a characteristic type of videos (ASMR videos). The present study examines whether this experience is indeed specific to these ASMR videos, or whether it can also be triggered by other types of videos, e.g. walking tour videos. A further goal was to investigate differences between ASMR-responders and ASMR-non-responders regarding their ASMR sensation and to compare ASMR and walking tour videos with regard to the elicitation of positive affect and state relaxation.

**Method:**

Two online assessments were carried out in two different predominantly student samples, one involving ASMR videos (n = 205) and the other one walking tour videos (n = 96). In both groups, ASMR experience, positive affect and state relaxation were assessed.

**Results:**

Compared to the walking tour video group, the ASMR-responders in the ASMR video group reported a pronounced ASMR sensation, higher state relaxation and higher positive affect scores. For ASMR-non-responders, lower scores in ASMR sensation, state relaxation and positive affect were revealed compared to the walking tour group. Without differentiating ASMR responder types, the ASMR group showed higher ASMR scores and lower positive affect scores compared to the walking tour group.

**Conclusions:**

Watching ASMR videos induced significantly more characteristic ASMR sensations compared to walking tour videos. Since ASMR videos typically include a simulated interaction of the video protagonist with the viewer and walking tour videos do not, the simulated interaction with the viewer might be one important factor for triggering ASMR. As the ASMR observer type (responder or non-responder) is crucial for benefitting from ASMR videos, future scientific evaluation of ASMR needs to consider this differention when evaluating effects of ASMR on mental heath associated domains.

## 1. Introduction

Autonomous Sensory Meridian Response (ASMR) is defined as a tingling sensation that is perceived on the skin (especially on the scalp and the back of the neck) and is usually associated with relaxation and well-being [1]. It has become increasingly popular in recent years, especially via internet platforms such as YouTube or TikTok, where videos specifically designed to trigger this characteristic ASMR experience often reach several million views. Despite this great popularity and the possible therapeutic potential of ASMR, a scientific evaluation of ASMR has hardly been conducted so far. First effects were recently demonstrated on aspects associated with mental health (inducing pleasant affect [1–3]; improving sleep quality [4]; inducing relaxation [3, 5]; alleviating state anxiety [6]). Furthermore, initial evidence suggests that ASMR experience also triggers physiological responses. Poerio et al. [2] showed that ASMR experience is associated with a significant reduction in heart rate (being associated with relaxation), whereas other studies describe an association between ASMR and–somewhat unexpectedly–increases in pupil diameter [7, 8] and skin conductance level [2] indicating increased arousal.

Furthermore, ASMR has been associated with social cognition, possibly due to the inherent social component of most ASMR videos with intimate situations often being shown, for example a role play depicting a doctor’s treatment. Social cognition is an umbrella term encompassing different socioemotional processes during social interactions such as emotion recognition, empathy and social problem solving [9]. For example, ASMR has been shown to be associated with empathy [10], which allows to infer and emotionally experience and/or respond to what other individuals feel, and with neural correlates in brain regions related to social cognition and self-awareness [11, 12]. This assumption is also reinforced by the fact that mindfulness–which has been associated with ASMR [13]–also seems to be related to social cognition [14].

It appears that ASMR does not occur in all individuals and that one can distinguish quite clearly between ASMR-responders and ASMR-non-responders. Different ASMR-responder rates have been provided in two studies: While Roberts et al. [15] state that 28 percent regard themselves as ASMR-responders, Swart et al. [16] report that 38 percent consider themselves to be responding to ASMR. Initial evidence suggests that ASMR experience is associated with specific personality characteristics, such as neuroticism [6, 17] or openness to experience [10, 17], the latter including greater openness towards aesthetic experiences and positive stimuli [18]. Furthermore, a link with mental health related traits such as mindfulness has been reported [13].

It is still relatively unclear how exactly ASMR videos must be designed to effectively trigger ASMR experiences. Specific characteristics that have been identified are lower-pitched, complex sounds without background music [19] and whispering, personal attention, and crisp sounds such as tapping fingernails [1].

Although there is some evidence linking ASMR to mental health related domains and social cognition, no randomized controlled trial (RCT) intervention study has been conducted so far showing that the repeated experience of ASMR improves positive affect, relaxation and social cognition. To adequately design such an RCT study, it would be important to compare ASMR videos to videos that are significantly less associated with ASMR experience and social cognition. Possibly, walking tour videos, another popular YouTube phenomenon, could fullfill these requirements, thus representing adequate videos to be used in comparison groups in future ASMR RCT intervention studies. In these walking tour videos, people film their walk through a certain location enabling the viewer to get a first person view (FPV) impression of the different locations through the eyes of the camera operator. Walking tour videos show some overlap with landscape videos that are also frequently uploaded to YouTube, with, for example, certain landscapes being filmed in high video resolution, but mostly either in a static manner or with the help of drones, lacking the characteristic walking element of walking tour videos. There are also soundscape nature videos uploaded to YouTube, with these videos being also presented in a rather static way and, unlike in walking tour videos, music is a crucial element. While there is at least some research on ASMR videos, to our knowledge, walking tour videos have not been scientifically evaluated until now. Just as for ASMR videos it is conceivable that they could improve well-being—for example by triggering positive affect linked to the wanderlust that drives many people and that often went unsatisfied especially during the COVID-19 pandemic. In walking tour videos, social cognition should play a minor role because the camera operator does not interact with other individuals or the viewer and the person walking is not visible in the video.

If walking tour videos moreover are indeed associated with less ASMR experience than ASMR videos, they might represent optimal videos to be used in comparison groups in future intervention studies that also assess social cognition. Thus, the main aim of the present study is to compare a group watching ASMR videos to a second group watching walking tour videos and to assess their self-reported ASMR experience including relaxation and positive affect. To our knowledge, this is the first ASMR study that explicitly also aims to evaluate non-ASMR videos that can be used in comparison groups in future ASMR intervention studies. When we speak of non-ASMR videos, we always mean videos that have not been explictly designed to trigger ASMR in other people, even though it must be kept in mind that in principle they can also unintentionally trigger ASMR. Previous studies also implemented non-ASMR videos, however without specially focusing on their evaluation. Cash et al. [20], Poerio et al. [2], Liu and Zhou [21], Smith et al. [12] as well as Fredborg et al. [22] used non-ASMR videos that were quite similar to the ASMR videos, e.g., they showed a person, often also facing the viewer. Swart et al. [23] asked the participants to submit ASMR videos that they knew for the control condition and edited them in a way that scrambled the original video. In a study by Valtakari et al. [7], the control condition included ASMR videos where the audio tracks were removed. In studies by Paszkiel et al. [24] and Wang et al. [25] no videos but silence, music or non-whispering voices were used as control conditions since these studies were limited to ASMR sounds and no video material was presented at all.

There is at least some reason to believe that non-ASMR videos have not been optimally selected so far. In the study by Liu and Zhou [21], the tingling sensation was more pronounced during the non-ASMR than during the ASMR videos although the other direction would have been expected. This underlines the importance of pre-evaluating comparison videos before the final video selection since videos that are not designed to trigger ASMR still might trigger ASMR unintentionally. Also, Valtakari et al. [7] report that at least some ASMR experiencers also had tingling sensations while watching the non-ASMR video. Cash et al. [20] found that while ASMR enthusiasts differ with regard to their ASMR-experience in each condition (ASMR clip vs. foil clip vs. music clip), especially naïve observers (mostly) do not. Fredborg et al. [22] did not describe a pre-evaluation of whether the ASMR stimuli they used actually trigger more ASMR experience than non-ASMR stimuli, but instead directly compared the electroencephalography (EEG) measures between the ASMR stimuli and non-ASMR stimuli groups. Similarly, Paszkiel et al. [24] and Wang et al. [25] did not assess the degree of ASMR experience for the ASMR stimuli and the non-ASMR stimuli separately. In all aforementioned studies no independent sample was recruited for the evaluation of the non-ASMR videos.

Evaluating non-ASMR videos, in particular those that focus less on a social component (in order to, for example, be able to detect potential effects of ASMR on social cognition in the future), will provide an important indication of whether the ASMR experience is significantly more specific to videos explicitly designed to trigger ASMR, or whether it can in principle–as some anecdotal evidence suggests–be triggered by videos with other content in an equally reliable manner. The comparison of ASMR videos with these non-ASMR videos could also generate further information about the characteristics that videos usually have in order to trigger ASMR.

Before pursuing the superordinate aim of the study, information about the validity of the ASMR construct will be obtained in this study by examing the association between the ASMR-responder / non-responder self-assessment and the reported ASMR experience. Furthermore, the assumption that due to the heterogeneity of the videos, differences in the effectiveness of ASMR triggering for the individual videos can be found, will be tested. Thus, the following three hypotheses will be examined in this study:

Hypothesis 1: In the ASMR video group, those who consider themselves to be ASMR-responders, report significantly higher ASMR scores in an ASMR questionnaire compared to those who do not consider themselves to be ASMR-responders.

Hypothesis 2: The individual ASMR videos are associated with different levels of ASMR experience. Moreover, the reported ASMR experience across the different videos is higher for ASMR-responders in comparison to non-responders.

Hypothesis 3: The ASMR video group reports a significantly more pronounced ASMR experience compared to the group that watched walking tour videos.

Exploratively, as part of the analyses conducted with regard to Hypothesis 3, it will also be examined whether a difference with regard to relaxation and positive affect can be observed between the two groups. Apart from the previously mentioned hypotheses, it will also be examined exploratively whether walking tour videos are indeed not perceived as containing a social component, thus suggesting that they might be appropriate to be used in a comparison group in future ASMR studies trying to reveal effects in terms of social cognition. Finally, a further ASMR-responder/non-responder rate will be provided to get additional information about the number of people who experience ASMR.

## 2. Methods

### 2.1. Participants

Participants were recruited for two independent groups: one watching ASMR (n = 205) and the other walking tour videos (n = 96). While for the ASMR group data were collected from December 2021 until May 2022, data collection for the walking tour group started two months later and ended at the same time as the ASMR study. Recruitment took place via newsletters and social media platforms, targeting mainly students from various German universities. Completing the study took about 1 hour in each group. Participants did not receive any remuneration for their participation. To keep the results as representative as possible, we avoided advertising within the ASMR or walking tour video community, for example via websites such as YouTube. To take part in the study, the participants should not have any neurological / psychological diseases, as assessed via self-report, they had to be able to speak German fluently and be at least 18 years old. Furthermore, they were required to use headphones while watching the videos. The demographic data of the respective samples (ASMR vs. walking tour group) are presented in Table 1. All participants gave informed written consent online to participate in the study.

**Table 1:** Demographic characteristics of the different study groups (ASMR vs. walking tour).

Characteristics	Group		Overall (n = 301)	Statistical values	df	p -values
	ASMR group (n = 205)	Walking tour group (n = 96)				
Age in years	M = 25.07 (SD = 7.20)	M = 24.76 (SD = 5.26)	M = 24.97 (SD = 6.63)	F = .15	1,299	n.s.
Gender	154 f/50 m/1 d	68 f/28 m/0 d	222 f /78 m/1 d	χ2 = 1.21	2	n.s.
Marital status	99 single/89 firm relationship/16 married/1 divorced or separated/0 widowed	55 single/35 firm relationship/6 married/0 divorced or separated/0 widowed	154 single/124 firm relationship/22 married/1 divorced or separated /0 widowed	χ2 = 2.49	3	n.s.
Highest completed level of education	0 no degree/0 certificate of secondary education/4 general certificate of secondary education/162 high school graduation/33 university degree/6 completed vocational training	0 no degree/0 certificate of secondary education /0 general certificate of secondary education/69 high school graduation/20 university degree /7 completed vocational training	0 no degree/0 certificate of secondary education/4 general certificate of secondary education/231 high school graduation/53 university degree/13 completed vocational training	χ2 = 6.03	3	n.s.
Occupational status	5 not employed or househusband or housewife/5 self-employed/32 employee or civil servant/162 pupil or student/0 unemployed /1 retired	2 not employed or househusband or housewife/2 self-employed/15 employee or civil servant/77 pupil or student/0 unemployed/0 retired	7 not employed or househusband or housewife/7 self-employed/47 employee or civil servant/239 pupil or student/0 unemployed/1 retired	χ2 = .55	4	n.s.

Note: d = non-binary; f = Female; m = Male; M = Mean; n.s. = not significant; SD = Standard deviation.

### 2.2. Procedure, stimulus material and assessments

#### 2.2.1 Procedure

In both groups (ASMR and walking tour) participants were expected to watch ten different three-minute videos. Before the videos were presented in an online questionnaire, in both groups demographic data (age, gender, marital status, highest completed level of education, and occupational status) were assessed. Then, sequentially, the respective videos were shown separately, with participants being asked not to continue until they had watched the entire video. All participants watched the videos in the same order. After the entire sequence of ten videos (ASMR vs. walking tour) was shown, the ASMR-15 [26]–a scientifically validated scale for measuring ASMR experience (see section 2.2.3)–and several other single items that are described in section 2.2.3 were presented to the participants, especially to assess ASMR experience, positive affect and state relaxation. The local ethics committee of the Ruhr University Bochum approved the study procedure.

#### 2.2.2 Stimulus material

For both the ASMR videos and the walking tour videos, permission for use in this study was obtained from the respective YouTubers. For the ASMR videos, care was taken to select ASMR videos that were as diverse as possible (e.g. gender, triggers) in order to gain a comprehensive impression of which components and which content of an ASMR video could be particularly reliably associated with ASMR experience. With respect to the ten different three-minute walking tour videos, care was taken to ensure that the walking tour videos depicted diverse locations. Besides paying attention to the diversity of the ASMR and walking tour videos, the ASMR and walking tour videos were randomly selected, but the final selection was also depending on the YouTuber’s consent to use the videos for our research.

**Fig 1:**
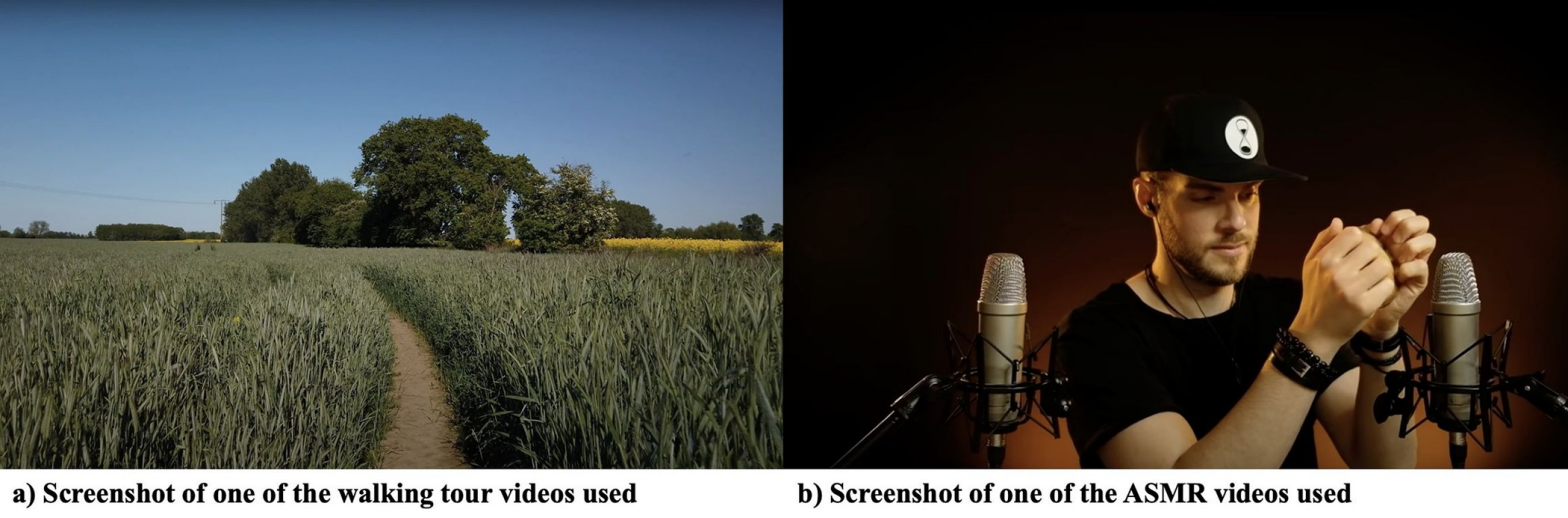
Presentation of a screenshot of a) a walking tour video (provided by Tony Kasten) and b) an ASMR video (provided by ASMR Zeitgeist) used in the framework of this study.

An overview of all ASMR and walking tour videos used in this study including the title of the videos, the YouTuber and the YouTube link can be found in Table 2. Furthermore, for a better immediate understanding of what an ASMR video and a walking tour video typically depict, we present a screenshot from one of the videos used in the respective video categories in Fig 1.

**Table 2:** List of all videos used in the respective group including YouTube link.

Group	Video number	Video title	YouTuber	YouTube-Link
ASMR	1	ASMR Hypnotic Trigger Mix to Help You Tingle, Sleep & Relax (Talking / No Talking)	asmr zeitgeist	https://www.youtube.com/watch?v=q-wkiB6n-38
ASMR	2	ASMR Best Triggers for Tingles (No Talking)	ASMR Justin	https://www.youtube.com/watch?v=lRKd-TIxCek
ASMR	3	ASMR Deutsch Flüstern: Arzt untersucht deine Augen und Ohren | Sehtest und Hörtest (ASMR Roleplay) [ASMR German Whispering: Doctor examines your eyes and ears | Eye test and hearing test (ASMR Roleplay)]	ASMR mit Kim	https://www.youtube.com/watch?v=N7y5F4aHycs
ASMR	4	ASMR Der BESTE Magier bringt Dich zum Einschlafen | Deutsch [ASMR The BEST magician puts you to sleep | German]	KennyK ASMR	https://www.youtube.com/watch?v=SB5dQ7JpfxQ
ASMR	5	ASMR deutsch | Hair Play Scalp Massage (Real Person) Brushing Neck Scratching Relaxing Two Girls	junebear asmr	https://www.youtube.com/watch?v=qKA40pbQ5wQ
ASMR	6	ASMR 20min kopfmassage (+ wind & bisschen brushing sounds) | emily asmr [ASMR 20min head massage (+ wind & a bit of brushing sounds) | emily asmr]	emily asmr	https://www.youtube.com/watch?v=LduSiVHrfIU
ASMR	7	Nature meets a male ASMRtist | DE	Nick Jameson ASMR	https://www.youtube.com/watch?v=XeO8Vdai248
ASMR	8	ASMR Deutsch Flüstern ✨ Positive Affirmation, Face Brushing & Personal Attention (German ASMR)	ASMR mit Kim	https://www.youtube.com/watch?v=-mXv3l7cRSk
ASMR	9	ASMR | Meine 10 besten TAPPING TRIGGER! [ASMR | My 10 best TAPPING TRIGGERS!]	DeutscheASMR	https://www.youtube.com/watch?v=T6RpiS4uh-Q
ASMR	10	ASMR Tapping Makes Good Sounds	RaffyTaphyASMR	https://www.youtube.com/watch?v=IEnVXQ8Sc_Y
Walking Tour	1	Walking Tour of Real MARRAKECH—Morocco Africa Video Walk【4K】MA	Wanna Walk	https://www.youtube.com/watch?v=hlCNJ5Qt83s
Walking Tour	2	Night Walking Tour of Times Square Midtown Manhattan, New York City 【4K】US	Wanna Walk	https://www.youtube.com/watch?v=eEaYrzhxiBY
Walking Tour	3	London Walk, REGENT STREET—Walking Tour【4K】GB	Wanna Walk	https://www.youtube.com/watch?v=ynbIuLhlY9Y
Walking Tour	4	Walking Tour of LISBOA, Portugal—Alfama Video Walk【4K】PT	Wanna Walk	https://www.youtube.com/watch?v=ABZOBi7nIC0
Walking Tour	5	Strand Spaziergang Ostsee im Winter, Schnee, Eis, Sonnenschein, Entspannung, virtual Jogging [Beach walk Baltic Sea in winter, snow, ice, sunshine, relaxation, virtual jogging]	SyncSouls: Entspannung, Musik, Heilung (Torsten Abrolat)	https://www.youtube.com/watch?v=KB-0e2VsKro
Walking Tour	6	Rio De Janeiro, BRAZIL—IPANEMA Beach, Walking Tour in RIO (Narrated) City Walks【4K】 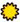 BR	Wanna Walk	https://www.youtube.com/watch?v=qADo6lZTcDU
Walking Tour	7	Entspannter Spaziergang Feldweg & Weizenfeld ∷ Natur ∷ Relaxen und Abschalten [Relaxed walk field path & wheat field ∷ Nature ∷ Relax and de-stress]	Waldgeflüster (Tony Kasten)	https://www.youtube.com/watch?v=rT2DehcWNp0
Walking Tour	8	Waldspaziergang im Herbst ∷ Naturgeräusche ∷ Kraft tanken ∷ Waldgeflüster [Forest walk in autumn ∷ Sounds of nature ∷ Recharge your batteries ∷ Whisper of the forest]	Waldgeflüster (Tony Kasten)	https://www.youtube.com/watch?v=4rdyMyZKBcg
Walking Tour	9	PRIMARK—Madrid, Spain (España) Walking Tour【5K】 ES	Wanna Walk	https://www.youtube.com/watch?v=xiLfJ0pQL1A
Walking Tour	10	BUENOS AIRES, Argentina—Heavy Thunderstorm (ASMR Umbrella City Rain Sound) WALKING TOUR【4K】☔AR	Wanna Walk	https://www.youtube.com/watch?v=yZl2E2Oz-60

#### 2.2.3 ASMR, positive affect and state relaxation assessment

The ASMR-15 is a 15-item questionnaire for which the authors report an internal consistency of α = .78 for the ASMR-15 total score [26]. The ASMR-15 comprises four different subscales: Sensation (five items, α = .72), Relaxation (three items, α = .74), Affect (three items, α = .74) and Altered Consciousness (four items, α = .82 [26]). The internal consistency of the ASMR-15 in the present study (calculated in the ASMR group) is very high (total α = .94, Sensation (α = .88), Relaxation (α = .89), Affect (α = .88) Altered Consciousness (α = .89)). Furthermore, an exploratory factor analysis confirmed that the four factor design of the ASMR-15 could be reproduced within our sample. For this study, the ASMR-15 was translated into German by our research team according to the translation and back translation method suggested by Brislin [27].

In addition to ASMR-specific items that attempt to assess the characteristic ASMR feeling (Sensation subscale), the ASMR-15 also includes items that assess more general aspects of mental health, such as the experience of positive affect and state relaxation (e.g. ‘I feel euphoric’, ‘I feel relaxed’). For the walking tour group, the introductory sentence of the ASMR-15 was modified so that the items subsequently referred to walking tour videos instead of ASMR-typical features. A (minimally adjusted) selection of five of the 15 ASMR-items was also presented after each individual video to get an idea of which of the ASMR / walking tour videos used were associated with the highest perceived ASMR experience (the exact items being: While watching the video… . 1. ’I experienced pleasant sensations’, 2. ’I experienced a pleasant tingling sensation on my skin’, 3. ’I experienced some kind of goose bumps on the back of my head’, 4. ’I felt relaxed’, 5. ’I felt joyful’, with the same answer options as presented in the ASMR-15). This selection was made because it includes the items that most accurately describe the core ASMR experience, i.e., whether pleasant sensations were experienced, whether these sensations appeared to resemble a tingling sensation on the skin, and whether goose bumps were experienced on the back of the head. Furthermore, this item selection was made since it also includes ASMR-nonspecific items related to positive affect and state relaxation while watching the videos, offering to analyse in this study not only the core ASMR experience, but also interesting mental health related variables (for each video, one item referred to affect, one item to relaxation and three items to the typical ASMR sensation being experienced on the skin).

In both groups, two items included questions on relaxation techniques (meditation, autogenic training, progressive muscle relaxation, massages, yoga), assessing whether certain techniques were already used regularly by the participants (e.g.: ‘Do you meditate regularly in your free time [several times a month]?’; answer options: yes / no) or whether they could imagine to engage in a certain relaxation technique (e.g., ‘Would you consider yourself to be a person who could engage in meditation?’; answer options: definitely yes / rather yes / rather no / definitely no / I do not know, because I have no experience with it). In addition, both groups included items assessing the familiarity of the respective phenomenon (ASMR in the ASMR video group vs. walking tour videos in the walking tour video group), i.e. whether the participant regularly watches ASMR videos / walking tour videos, and whether the participant would recommend viewing ASMR videos or walking tour videos to friends and acquaintances. In addition, participants were asked to estimate whether ASMR videos / walking tour videos could help improve mental health ("Do you think watching ASMR / walking tour videos regularly could help improve mental health?"; answer options: yes / no) and whether each phenomenon had the potential to improve various socio-cognitive domains (emotion recognition, perspective taking, social behavior; e.g. the item used for emotion recognition was: "Do you think watching ASMR / walking tour videos on a regular basis might help improve your ability to recognize other people’s feelings?"; answer options: yes / no).

In the walking tour group, questions referring to potential social aspects of walking tour videos were added to find out whether walking tour videos, unlike ASMR videos, are perceived to be deprived of a social component (1. ‘While watching the walking tour videos, did you get the feeling that you yourself were in the place shown?’, 2. ‘Were you aware of the presence of the camera operator while watching the walking tour videos?’, 3. ‘While watching the walking tour videos, did you have the feeling of taking someone else’s perspective?’, 4. ‘While watching the walking tour videos, did you feel like you were witnessing the events in the videos from your own perspective?’, 5. ‘Did you have the feeling of being alone while watching the walking tour videos?’).

Only in the ASMR group, participants were asked to self-assess their ability to experience ASMR after watching all ASMR videos as part of this questionnaire (‘Would you consider yourself a person who experiences ASMR?‘; answer options: definitely yes / rather yes / rather no / definitely no). The walking tour questionnaire instead asked whether participants watch ASMR videos regularly and whether they expect themselves to be able to engage with them.

### 2.3. Data analysis

SPSS 28 was used for data analysis. Chi square and univariate analyses of variance were calculated to examine whether the samples for the respective groups (ASMR vs. walking tour) were comparable regarding sample characteristics and to compare ASMR-responders vs. ASMR-non-responders with regard to their ASMR experience. Repeated-measures analyses of variance (within subjects) and descriptive analyses were used to examine how strongly each ASMR video was associated with ASMR / affect / relaxation experience. Group (ASMR versus walking tour videos) and observer type (responder vs. non-responder) were added as between subjects factor. For the categorization of ASMR-responders, the response options “definitely yes” and “rather yes” to the question of whether participants would consider themselves a person who in general experiences ASMR were combined. Similarily, the answer options “rather no” and “definitively no” were combined to indicate ASMR-non-responders. Multivariate and univariate analysis of variance were calculated to ascertain whether the groups of the respective samples differed in terms of their ASMR / affect / relaxation experience (between subjects analysis).

## 3. Results

A univariate analysis of variance was conducted to compare the mean ASMR-15 total scores between the groups of participants that considered themselves as definitely, rather, rather not or definitely not experiencing ASMR. A significant difference between the groups was revealed (F(3, 201) = 104.14, p < .001, η^2^_p_ = .61). Regarding the groups’ mean scores (see Table 3) it becomes evident that the more the participants considered themselves to be an ASMR-responder, the higher their total scores appeared to be. Consequently, in accordance with the expectations, the participants’ ASMR-responder self-assessment did significantly differ with regard to their ASMR-15 scores, thus confirming Hypothesis 1.

**Table 3:** Differences in ASMR-15 total scores for self assessed ASMR-responders and ASMR-non-responders.

Would you consider yourself a person who experiences ASMR?	Mean ASMR-15 total scores	Standard Deviation (SD)	N
1 = definitely yes	58.50	5.52	30
2 = rather yes	53.58	9.29	57
3 = rather no	39.07	10.90	70
4 = definitely no	26.17	9.53	48

To determine whether the ASMR videos differed in their ability to trigger ASMR, a repeated-measures analysis of variance with observer type (responder vs. non-responder) being added as a between subjects factor was calculated. The dependent variable was the total score of the three items related to the ASMR sensation which were presented after each video (excluding the items which can rather be seen as concomitant effects of ASMR, namely positive affect and relaxation). It was found that the ten videos differed significantly in terms of the reported ASMR experience (F(9, 169) = 15.75, p < .001, η^2^_p_ = .46). A significant difference between the videos was also observed in terms of the positive affect item (F(9, 182) = 19.15, p < .001, η^2^_p_ = .49) and the relaxation item (F(9, 180) = 19.18, p < .001, η^2^_p_ = .49) presented after each video. The respective means and standard deviations are presented in Table 4. Considering the ASMR experience more broadly, not only taking into account the core ASMR sensation items but including also the items assessing the concomitant effects of relaxation and positive affect in an ASMR total score, it again becomes apparent that the ASMR videos differ significantly from each other in terms of their ability to trigger ASMR (F(9, 169) = 15.75, p < .001, η^2^_p_ = .46).

**Table 4:** Mean values regarding ASMR experience, affect and relaxation for the individual ASMR videos.

Video (n = 191)	ASMR experience sum score mean (SD)	Affect	Relaxation
		mean (SD)	mean (SD)
1 (ASMR)	8.23 (3.06)	3.21 (1.07)	3.58 (1.34)
2 (ASMR)	7.57 (3.49)	2.86 (1.20)	3.01 (1.38)
3 (ASMR)	6.52 (3.46)	2.41 (1.26)	2.74 (1.46)
4 (ASMR)	6.27 (3.18)	2.51 (1.20)	2.63 (1.34)
5 (ASMR)	7.38 (3.43)	2.85 (1.16)	3.20 (1.31)
6 (ASMR)	7.28 (3.77)	2.64 (1.21)	2.84 (1.38)
7 (ASMR)	7.90 (3.16)	3.48 (1.25)	3.68 (1.32)
8 (ASMR)	6.80 (3.42)	2.50 (1.20)	2.79 (1.44)
9 (ASMR)	6.50 (3.09)	2.54 (1.19)	2.74 (1.34)
10 (ASMR)	7.82 (3.34)	3.06 (1.16)	3.34 (1.31)

**Fig 2:**
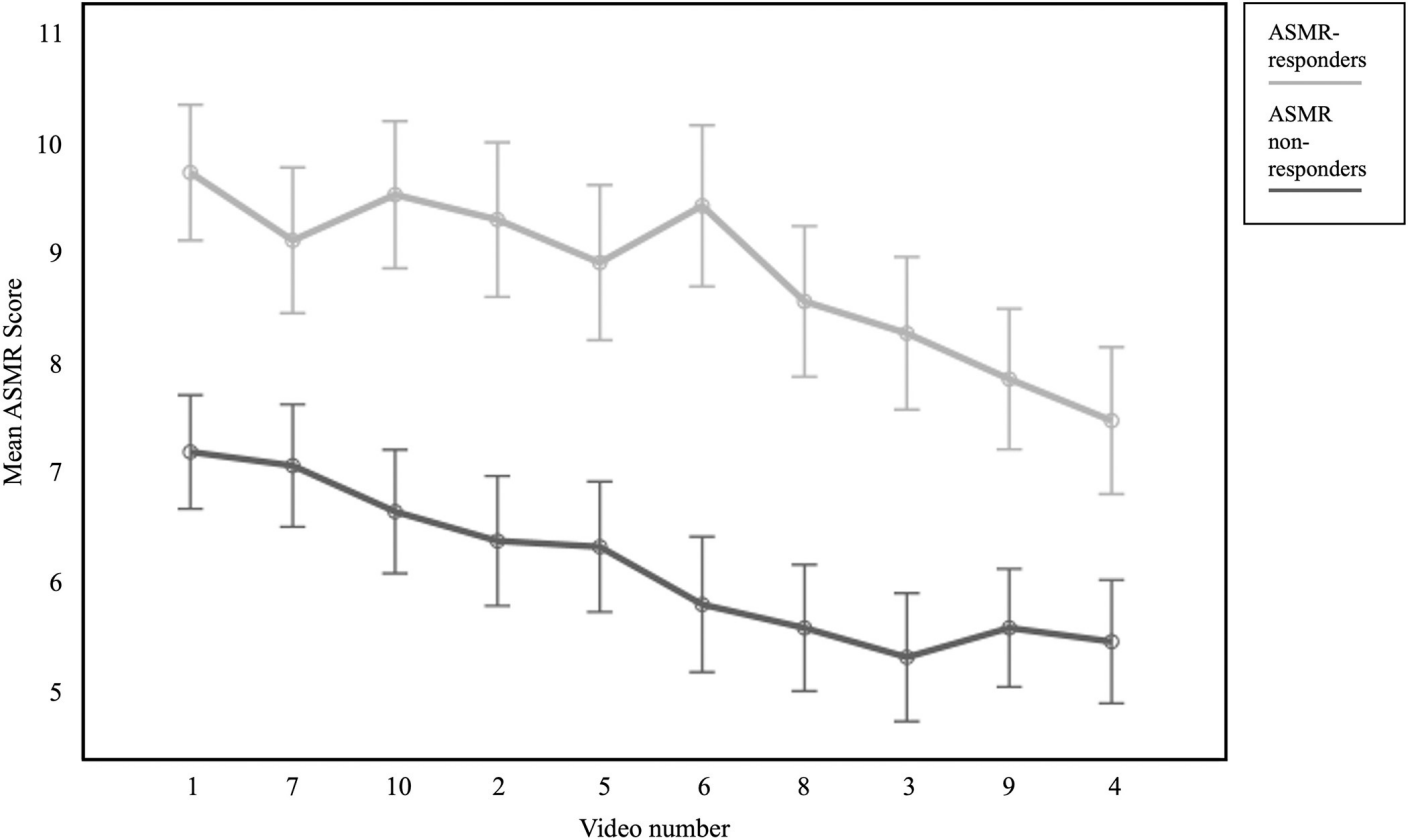
Average ASMR scores for each of the ten ASMR videos subdivided for ASMR-responders and ASMR non-responders with the videos being sorted by the amount of ASMR experience (main effects of video and observer type).

Moreover, there was a main effect of the observer type with responders reporting increased ASMR experience across all ten ASMR videos (F(1, 189) = 78.88, p < .001, η^2^_p_ = .29). However, there was no significant interaction between observer type (responder vs. non-responder) and reported ASMR experience across the individual videos (F(9, 181) = 1.67, p = .098, η^2^_p_ = .08). For a better overview, Fig 2 graphically shows the mean ASMR scores of each video with the distinction between ASMR-responders and non-responders. The videos are ranked starting with the video with the highest reported ASMR sensation to the lowest ASMR sensation (across responders and non-responders). The results are consistent with Hypothesis 2 as the ASMR experience is different dependent on the individual video and as there are significant differences between responders and non-responders across the ASMR videos (main effects of video and observer type).

With respect to differences between the ASMR group and the walking tour group concerning ASMR experience (Hypothesis 3), as a first step the total score of the ASMR-15 was compared between both groups conducting a univariate analysis of variance. No significant difference was found with regard to the ASMR-15 total score. In an additional analysis of variance, participants who defined themselves as ASMR-responders were analyzed separately from participants who defined themselves as ASMR-non-responders and contrasted to the walking tour video group. The mean ASMR-15 scores were 55.31 (SD = 8.72) for the ASMR-responders, 32.75 (SD = 11.39) for the ASMR-non-responders and 41.42 (SD = 12.10) for the walking tour group. This analysis showed significant differences between the three groups (F(2, 274) = 96.89, p < .001, η^2^_p_ = .41), thus indicating that more ASMR experience is reported for ASMR-responders when compared to participants of the walking tour group.

Without differentiating between ASMR-responders and ASMR-non-responders, an additional MANOVA was conducted including all four ASMR-15 subscales as dependent variables. The results revealed significant differences between both groups (F(4, 272) = 9.28, p < .001, η^2^_p_ = .12). With respect to the Sensation subscale (F(1, 275) = 7.36, p = .007, η^2^_p_ = .03) the means indicated in line with the expectations significantly more ASMR typical sensation (e.g. tingling experience on the skin) in the ASMR group (mean = 2.76, SD = 1.15) in comparison to the walking tour group (mean = 2.38, SD = 1.00). Furthermore, it was revealed that affect-related scores (Affect subscale of the ASMR-15; F(1, 275) = 4.74, p = .030, η^2^_p_ = .02) are even higher in the walking tour group (mean = 3.11, SD = .95) than in the ASMR group (mean = 2.80, SD = 1.17). With respect to the subscales Relaxation and Altered Conciousness, no differences between the groups could be observed. An additional MANOVA with distinguishing between ASMR-responders, ASMR-non-responders and walking tour group participants again revealed a main effect (F(8, 544) = 26.30, p < .001, η^2^_p_ = .28). As univariate analyses show, there were significant differences for the subscales Sensation (F(2, 274) = 81.52, p < .001, η^2^_p_ = .37), Relaxation (F(2, 274) = 61.67, p < .001, η^2^_p_ = .31), Affect (F(2, 274) = 75.89, p < .001, η^2^_p_ = .36) and Altered Consciousness (F(2, 274) = 33.10, p < .001, η^2^_p_ = .20). In all cases, the values were highest for the ASMR-responder group (Sensation: mean = 3.71, SD = 0.74; Relaxation: mean = 4.32, SD = 0.73; Affect: mean = 3.73, SD = 0.82; Altered Consciousness: mean = 3.16, SD = 1.12) in comparison to ASMR-non-responders (Sensation: mean = 2.09, SD = 0.88; Relaxation: mean = 2.78, SD = 1.19; Affect: mean = 2.14, SD = 0.90; Altered Consciousness: mean = 1.88, SD = 0.88) and the walking tour group (Sensation: mean = 2.38, SD = 1.00; Relaxation: mean = 3.57, SD = 0.77; Affect: mean = 3.11, SD = 0.95; Altered Consciousness: mean = 2.37, SD = 1.19).

The exploratory analyses concerning the social component of the walking tour videos revealed mixed results. The absence of a social component in walking tour videos seems to be supported by the fact that the majority of participants felt they were on their own at the location shown (10.4 percent strongly agreed, 53.1 percent rather agreed). However, this is contradicted by the fact that the vast majority of participants reported noticing the presence of the camera operator (20.8 percent strongly agreed, 45.8 percent rather agreed), did not have the feeling of being alone while watching the videos (8.3 percent strongly disagreed, 46.9 percent rather disagreed) and had the impression of taking the perspective of another person (18.8 percent strongly agreed, 53.1 percent rather agreed). Interestingly, this answer pattern concerning perspective taking changes if the question is framed somewhat differently, asking whether the events are experienced from the participants’ own perspective. Then the majority of the participants in fact state that they perceive the events from their own perspective (9.4 percent strongly agreed, 57.3 percent rather agreed). Overall, it must be nevertheless concluded that, contrary to our expectations, the statistical evaluation indicates that a social component is perceived in walking tour videos.

When it comes to estimating an ASMR responder rate evaluating how many participants report that they experience ASMR, 14.6 percent of the participants state that they “definitely” experience ASMR, while 27.8 percent state that they are “likely” to do so. Thus, the ASMR responder rate determined in this study varies from 14.6 percent to 42.4 percent depending on whether or not one includes participants who are not entirely sure. Presumably, the actual responder rate will be in the middle of these two values, thus approximately matching the responder rate determined by Roberts et al. [15].

## 4. Discussion

In this study, further evidence was gathered for the validity of the ASMR construct since subjective ASMR responder categorization is associated with the ASMR experience questionnaire data (Hypothesis 1). Moreover, the ASMR-15 scores of the subscale Sensation differ between the ASMR and the walking tour group, suggesting that the core ASMR sensation (tingling sensation on the neck and scalp) is indeed more likely to be triggered by ASMR videos than by walking tour videos (Hypothesis 3). When distinguishing between ASMR-responders and ASMR-non-responders, the effects for ASMR-responders are even more pronounced, since ASMR-responders have higher scores on the ASMR-15 total score as well as on every ASMR-15 subscale compared to the participants in the walking tour group.

Since walking tour videos are associated with less ASMR experience compared to ASMR videos, they might be appropriate to be used in comparison groups in future RCT studies. If a future RCT is, however, designed to investigate effects on social cognition, this study provides a hint that walking tour videos might, contrary to our expectation, not be appropriate videos for a comparison group, since they appear to exhibit a meaningful social component. Future studies should investigate whether this social component is perceived to be significantly lower compared to ASMR videos. If this is the case, it could be argued that despite certain social components inherent in walking tour videos, these kind of videos also represent adequate non-ASMR videos to be used in an RCT study in terms of an investigation of effects of ASMR on social cognition.

This social component in walking tour videos does not involve interaction of the video protagonist with the viewer, as this interaction only exists in ASMR videos. One possible reason for the fact that more ASMR experience is reported with regard to ASMR videos compared to walking tour videos could therefore be the lack of interaction with the viewer in walking tour videos. However, other reasons are also possible, such as the deliberate creation of an intimate, caring atmosphere, which is also not created in walking tour videos.

Importantly, the self categorisation as ASMR-responder or ASMR-non-responder is to be considered since self-rated ASMR-responders score higher not only in ASMR-experience, but also in state relaxation and positive affect than participants in the walking tour group. This means that it is absolutely crucial to take into account in future studies whether someone experiences ASMR or not, as the effects are significantly different when this distinction is taken into account. Although further studies are needed, an implication based on the anecdotal evidence of this important finding could be that ASMR videos are very promising in terms of well-being if one is able to experience ASMR, whereas people who do not experience ASMR might not benefit from ASMR videos to the same degree. However, this might not be true in general, since for example in the study by Poerio et al. [2] it was shown that the decrease in heart rate occurred in ASMR-responders as well as in ASMR-non-responders.

As a side effect of this study, it became apparent that besides ASMR videos walking tour videos might have unprecedented potential, as positive affect could be prompted to a higher degree during these videos than during the ASMR videos when comparing both groups without differentiating ASMR-responders and ASMR-non-responders. It could well be that effects of travel on psychological well-being, which can be shown for example when comparing well-being during a free evening at home and during a trip to another place [28], can also be generated to a certain extent when the trip is only simulated instead of actually being undertaken. To further evaluate the possible potential of walking tour videos, future research should investigate whether they also induce higher positive affect when compared not only to ASMR videos, but also to other video categories. It would for example be possible to compare walking tour videos to other video categories popular on YouTube, such as the aforementioned walking tour related landscape or soundscape videos or unrelated video content such as cooking videos or tutorials.

As expected there was also confirmatory evidence for Hypothesis 2 in our study suggesting differences in the potential of the videos to trigger ASMR. Although this can only be done in a speculative and descriptive manner at this point, a few salient features can be identified based on common features of videos that are particularly strongly associated with ASMR experiences, thus allowing some, albeit very limited, anecdotal evidence to be derived in relation to the question of which characteristics of ASMR videos are essential in order to trigger ASMR.

In three of the five videos most strongly associated with ASMR, tapping is involved as a trigger. Within the three videos most associated with ASMR, the two YouTubers with the most subscribers within the questionnaire used are represented (ASMR Zeitgeist & RaffyTaphy ASMR). It is possible that these YouTubers have special individual (personality) characteristics or are perceived as particularly likeable, which may explain their popularity as well as the increased ASMR values associated with their videos. Since video 7 is one of the three videos most associated with ASMR but without showing a person behind the camera, it is suggested that the typical ASMR video setting with a person present in front of the camera is definitely not mandatory to create an ASMR experience. Also, the good performance of this video contradicts the assumption described by Barratt et al. [19] that background music inhibits ASMR experience.

It is interesting to note that the ASMR experience is strikingly different across all ten videos between ASMR-responders and ASMR-non-responders. On the other hand, it is also interesting that the ASMR experience of ASMR-non-responders and ASMR-responders follows a very similar pattern across the videos (see Fig 2). The pattern is decreasing very similarily across the ten videos in both ASMR-responders and non-responders suggesting that the same pattern of ASMR experience is elicted by the individual videos although the intensity (or level) of the reported ASMR experience differs.

In this study, several limitations emerged, which will be discussed at this point. One important limitation is the difference in sample sizes between the two groups, especially being due to different starting points of both online studies. Also, the participants’ mean age (24.97) was rather low. Thus it remains unclear whether the ASMR-related (and walking tour-related) results–for example the provided ASMR-responder rate–are also applicable to an older sample. Furthermore, considerably more women were recruited than men with possible gender effects not having been evaluated in this study. It might also be the case that the ratio between ASMR-responders and ASMR-non-responders differs in the general population, as those who know ASMR could be more inclined to take part in an ASMR-related study, thus overestimating the actual percentage of ASMR-responders. Additionally, participants were classified as either ASMR-responders or non-responders based on subjective self-reports, although using the ASMR‐Experience Questionnaire (AEQ [16])–which is significantly longer than the ASMR-15 and was therefore not included in this study–would allow a more accurate determination of whether someone is an ASMR-responder.

Another limitation arises from the approach of assessing positive affect and state relaxation only as a part of the ASMR-15. Using separate affect and relaxation questionnaires would have provided more substantial data, but would, on the other hand, also have lengthened the respective questionnaires (that were already comprehensive due to the several videos included) to an extent that seemed inappropriate for the aims of this study. In addition, only five items were included to compare the individual videos with each other in terms of their ability to trigger ASMR, although it would have been much more meaningful to compare, for example, an ASMR-15 total score for each video. However, this was not possible, as otherwise the questionnaire would have been too long for a feasible implementation. Furthermore, only the walking tour questionnaire comprised questions related to the social component within the videos, although it could have been useful to add these questions to the ASMR questionnaire as well to be able to conduct group comparisons. This shortcoming is related to the fact that it was decided several months after the ASMR questionnaire was conceived to evaluate also walking tour videos and not only ASMR videos.

It should also be mentioned that the video resolution of some videos was rather poor, thereby possibly diminishing effects that would have emerged during a high definition ASMR and walking tour experience. Moreover, even though it was ensured that videos with diverse characteristics were used in each group, some types of videos were excluded, such as mukbang videos (popular videos of people eating that are also associated with ASMR), or walking tour videos with verbal comments. In addition, the various videos were not comprehensively pre-evaluated.

Despite these limitations, this study was able to generate further evidence regarding the validity of the ASMR construct by showing that the self-assessment of being an ASMR-responder is consistent with ASMR questionnaire data and that ASMR can be triggered more reliably by corresponding ASMR videos than by walking tour videos. ASMR appears to be a clearly circumscribed, subjectively unambiguous perceptual construct that can only be triggered by specific stimuli. In this study watching ASMR videos was related to significantly more positive affect and state relaxation for ASMR-responders in the ASMR group compared to participants watching walking tour videos in another group. Thus future studies should definitely further address the potential of ASMR, e.g., via conducting a comprehensive intervention study that compares the regular watching of ASMR videos with the watching of non-ASMR videos such as walking tour videos also paying attention to the differentiation between ASMR-responders and non-responders. This way, it would be possible to further specify whether ASMR videos have more benefits on mental health related outcomes such as positive affect and state relaxation when compared to other interventions (e.g. other video content).
